# Calcineurin is required for *Candida glabrata* Pdr1 transcriptional activation

**DOI:** 10.1128/mbio.02416-23

**Published:** 2023-11-09

**Authors:** Bao Gia Vu, Lucia Simonicova, W. Scott Moye-Rowley

**Affiliations:** 1 Department of Molecular Physiology and Biophysics, Carver College of Medicine University of Iowa, Iowa City, Iowa, USA; Universidade de Sao Paulo, Ribeirao Preto, Sao Paulo, Brazil

**Keywords:** *Candida glabrata*, fluconazole, antifungal resistance, transcriptional regulation, Pdr1

## Abstract

Fluconazole is one of the most commonly used antifungals today. A result of this has been the inevitable selection of fluconazole-resistant organisms. This is an especially acute problem in the pathogenic yeast *Candida glabrata*. Elevated minimal inhibitory concentrations for fluconazole in *C. glabrata* are frequently associated with substitution mutations within the Zn2Cys6 zinc cluster-containing transcription factor-encoding gene *PDR1*. These mutant Pdr1 regulators drive constitutively high expression of target genes like *CDR1* that encodes an ATP-binding cassette transporter thought to act as a drug efflux pump. Exposure of *C. glabrata* to fluconazole induced expression of both Pdr1 and *CDR1*, although little is known of the molecular basis underlying the upstream signals that trigger Pdr1 activation. Here, we show that the protein phosphatase calcineurin is required for fluconazole-dependent induction of Pdr1 transcriptional regulation. Calcineurin catalytic activity is required for normal Pdr1 regulation, and a hyperactive form of this phosphatase can decrease susceptibility to the echinocandin caspofungin but does not show a similar change for fluconazole susceptibility. Loss of calcineurin from strains expressing two different gain-of-function forms of Pdr1 also caused a decrease in *CDR1* expression and increased fluconazole susceptibility, demonstrating that even these hyperactive Pdr1 regulatory mutants cannot bypass the requirement for calcineurin. Our data implicate calcineurin activity as a link tying azole and echinocandin susceptibility together via the control of transcription factor activity.

**IMPORTANCE:**

Drug-resistant microorganisms are a problem in the treatment of all infectious diseases; this is an especially acute problem with fungi due to the existence of only three major classes of antifungal drugs, including the azole drug fluconazole. In the pathogenic yeast *Candida glabrata*, mutant forms of a transcription factor called Pdr1 are commonly associated with decreased fluconazole susceptibility and poor clinical outcomes. Here, we identify a protein phosphatase called calcineurin that is required for fluconazole-dependent induction of Pdr1 transcriptional activation and associated drug susceptibility. Gain-of-function mutant forms of Pdr1 still required the presence of calcineurin to confer normally decreased fluconazole susceptibility. Previous studies showed that calcineurin controls susceptibility to the echinocandin class of antifungal drugs, and our data demonstrate that this protein phosphatase is also required for normal azole drug susceptibility. Calcineurin plays a central role in susceptibility to two of the three major classes of antifungal drugs in *C. glabrata*.

## OBSERVATION

Pdr1 is a central regulator of fluconazole susceptibility in *Candida glabrata* and activates the expression of the ATP-binding cassette transporter-encoding gene *CDR1* (reviewed in reference 1). We have used a tandem affinity purification tagged form of Pdr1 to recover proteins that co-purify with this transcription factor. These data will be presented elsewhere, but one of the proteins of interest that we recovered was the catalytic subunit of the protein phosphatase calcineurin (Cna1) (reviewed in reference 2). Cna1 is well-established to control susceptibility to the echinocandin class of antifungal drugs via its activation of the Crz1 transcription factor (reviewed in reference 3). Cna1 was also linked to fluconazole susceptibility as *cna1*Δ strains exhibited increased azole drug susceptibility (4), although the underlying mechanism in *C. glabrata* has remained unknown. Additionally, extensive genetic analyses have suggested the possibility that calcium and calcineurin are associated with Pdr1 regulation (5–7). Here, we provide evidence that calcineurin is required for transcriptional activation by Pdr1.

To confirm the Pdr1:Cna1 association we detected by co-purification, we epitope tagged the *CNA1* gene with the insertion of two FLAG epitopes at the Cna1 carboxy-terminus to produce the Cna1-2× FLAG protein. This protein retained normal function, and we prepared whole-cell protein extracts from strains expressing wild-type or two different gain-of-function (GOF) forms of Pdr1. Pdr1 was immunoprecipitated from these strains, and associated Cna1 was detected by immunoblotting using an anti-FLAG antibody (Fig. S1). Cna1 was found to associate with all the forms of Pdr1 we examined. These data confirm that Cna1 is associated with Pdr1 *in vivo*, although we do not yet know if this represents a direct or indirect association.

**Fig 1:**
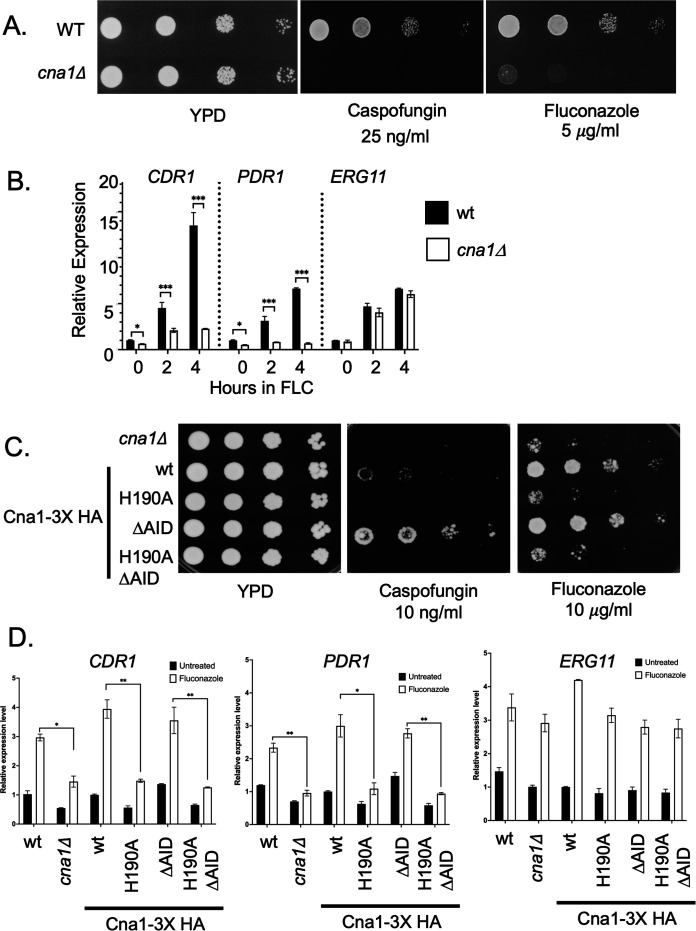
Calcineurin is required for fluconazole-induced *CDR1* and *PDR1* expression. (A) Isogenic wild-type and *cna1*Δ strains were grown to mid-log phase and serially diluted on 1% yeast extract, 2% casein peptone, and 2% dextrose anhydrous powder (YPD) medium containing the indicated concentrations of caspofungin and fluconazole for 24 hours. (B) Wild-type and *cna1*Δ strains were grown to mid-log phase and then treated with 20 µg/mL fluconazole for the indicated times on the X axis. (C) A strain lacking the *CNA1* gene (*cna1*Δ) was transformed with low-copy-number plasmids expressing the indicated forms of Cna1 as 3× HA-tagged proteins: the wild-type protein (wt), a catalytically inactive mutant protein (H190A), a constitutively active phosphatase mutant (ΔAID), or a double mutant (H190A ΔAID). Transformants were grown to mid-log phase, and serial dilutions were spotted on plates containing the indicated media for 36 hours. (D) The transformants expressing the indicated forms of Cna1, along with wild-type and isogenic *cna1*Δ control strains, were grown to mid-log phase, left untreated, or challenged with fluconazole as above and then assayed for levels of *CDR1*, *PDR1*, and *ERG11* mRNA using reverse transcription-quantitative PCR (RT-qPCR).

Having established that Cna1 was associated with Pdr1 *in vivo*, we analyzed the effect of Cna1 on Pdr1-dependent transcriptional activation by comparing isogenic wild-type and *cna1*Δ cells. We reproduced the published phenotypes caused by loss of Cna1 (4) as a *cna1*Δ strain exhibited a pronounced increase in susceptibility to both caspofungin and fluconazole (Fig. 1A). Importantly, the normal level of fluconazole-induced transcriptional activation of both *PDR1* and *CDR1* genes was lost in the *cna1*Δ strain (Fig. 1B). Additionally, the basal expression of both of these genes was reduced in the absence of Cna1. These data argue that the effect of Cna1 on fluconazole susceptibility comes about by stimulating the function of the Pdr1 transcription factor. Expression of the gene (*ERG11*) encoding the fluconazole target enzyme was unaffected by the loss of calcineurin activity. The impact of calcineurin on the expression of Cdr1, Pdr1, and Erg11 was also seen by western-blot analysis using antibodies directed against each of these proteins (Fig. S2A and C). Normal calcineurin activity is required for wild-type and fluconazole-induced expression of *CDR1* and *PDR1* but dispensable for *ERG11*.

We took advantage of the extensive characterization of Cna1 (discussed in reference 2) and tested two different mutant forms of this factor to assess the effect on Pdr1 transcriptional activation. We prepared a catalytically inactive mutant derivative (H190A Cna1) and a constitutively active form of Cna1 that lacks the autoregulatory inhibitory domain (AID) that would normally restrain phosphatase activity (reviewed in reference 8). These mutations were introduced into a *cna1*Δ strain and tested for the ability to complement the caspofungin and fluconazole hypersusceptibility of this strain as well as to correct the defective fluconazole induction of *PDR1* and *CDR1* transcription.

The presence of the ΔAID Cna1 protein led to decreased caspofungin susceptibility as has been seen for ion stress (9) but only restored fluconazole susceptibility to normal levels (Fig. 1C). To our knowledge, the decreased caspofungin susceptibility of the ΔAID Cna1 protein has not been reported before. Loss of the catalytic histidine residue from both forms of Cna1 led to the loss of complementing activity. While calcineurin activity is limiting for susceptibility to caspofungin, only normal levels are required for wild-type fluconazole susceptibility. This is supported by levels of fluconazole-inducible transcription of both *CDR1* and *PDR1* as the loss of the catalytic histidine blocked the drug-inducible expression of both these genes, and the loss of the AID restored normal levels of expression (Fig. 1D). Note that the effects of calcineurin impact the Pdr1/*CDR1* axis of fluconazole susceptibility but not *ERG11* as the response of the *ERG11* gene to fluconazole was unaffected by alterations in calcineurin activity.

Calcineurin activity requires both a catalytic (*CNA1*) and a regulatory subunit (*CNB1*). To ensure the regulatory subunit was also involved in the control of *CDR1* and *PDR1* expression, we prepared a *cnb1*Δ strain. To confirm the specificity of the effect of loss of calcineurin activity, we also generated a strain lacking the *PPT1* gene that encodes a Cna1-related protein serine/threonine phosphatase (10). Loss of *CNB1* led to an increase in fluconazole susceptibility similar to that caused by the *cna1*Δ allele (Fig. 1A), while loss of *PPT1* had no significant effect on this phenotype (Fig. S3A). We analyzed Cdr1, Pdr1, and Erg11 expression by western blotting (Fig. S3B and C). As the fluconazole phenotype suggested, the *cnb1*Δ strain did not induce either Cdr1 or Pdr1, while fluconazole induction was normal for the *ppt1*Δ strain. Erg11 expression was unaffected by the loss of either gene.

We also probed the effect of the immunosuppressive drug FK506 which is well-established as a potent inhibitor of calcineurin activity (11). The addition of 1 µM of FK506 led to a striking increase in the level of susceptibility to both fluconazole and caspofungin in wild-type cells (Fig. S4A). Importantly, the presence of FK506 completely blocked the fluconazole-dependent induction of *PDR1* transcription (Fig. S4B) as well as the increased levels of Pdr1 protein (Fig. S4C and D), while no significant effect was seen on the expression of *ERG11*.

**Fig 2:**
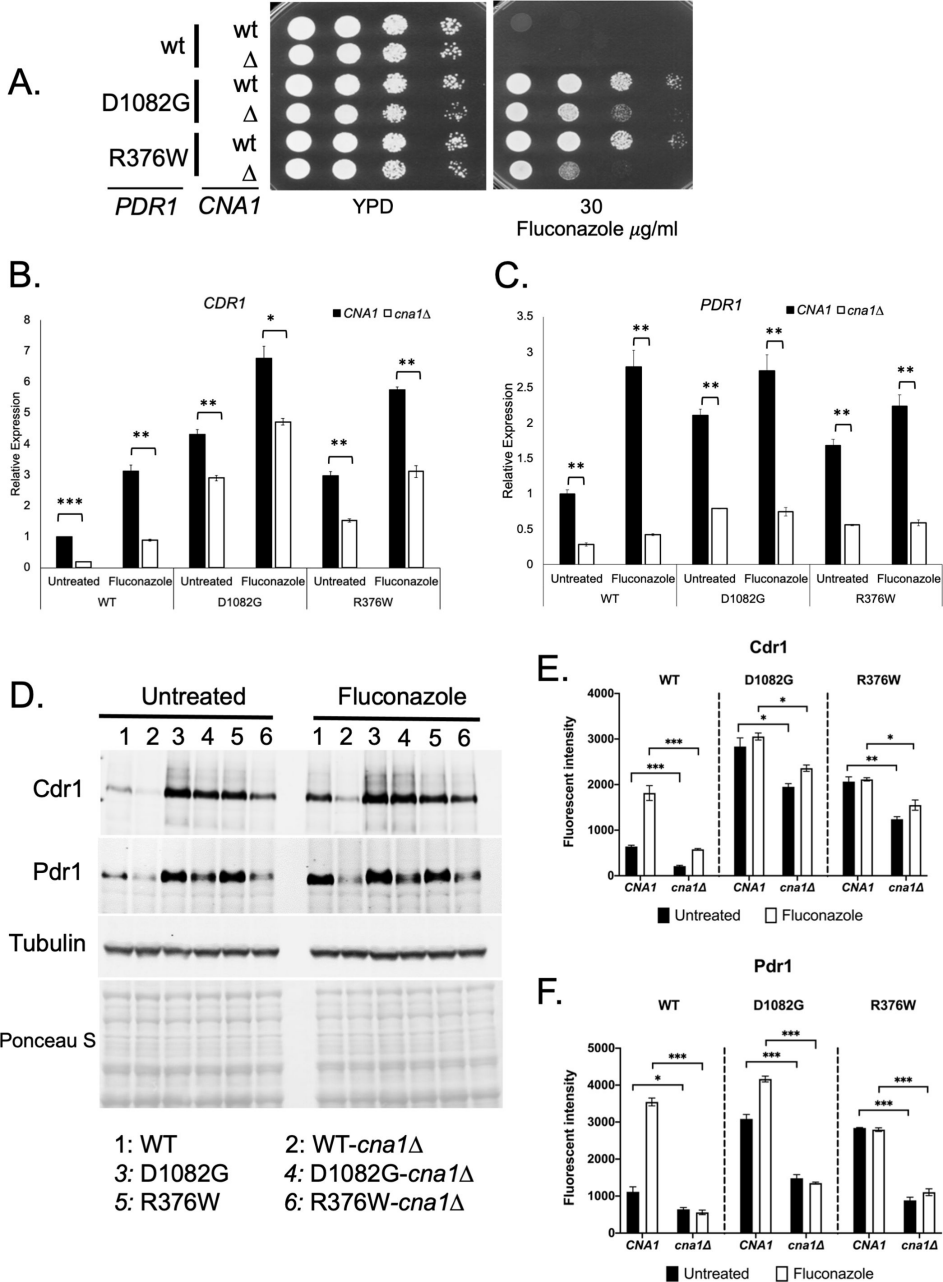
Calcineurin is required for normal activity of GOF forms of Pdr1. (A) Isogenic strains containing or lacking the *CNA1* gene and expressing the indicated forms of Pdr1 were grown to be tested for susceptibility to elevated level of fluconazole indicated as described before. (B) The strains listed above were tested for the level of untreated and fluconazole-inducible transcription of *CDR1* and *PDR1* by RT-qPCR as above. (C) Whole-cell protein extracts were prepared from the strains treated as in panel B and analyzed by western blotting using polyclonal antibodies directed against Cdr1 or Pdr1 and a mouse monoclonal that detects tubulin protein to ensure equal loading. The transferred proteins were also stained by Ponceau S after western blotting to confirm equivalent transfer and protein extraction. Quantitation of the western blot signals for Cdr1 (**E**) and Pdr1 (**F**) is also shown in the respective panels.

Having established that calcineurin was required for both wild-type and fluconazole-induced levels of Pdr1 function, we tested the role of this phosphatase on two different GOF alleles of *PDR1*. We disrupted the *CNA1* gene in three isogenic backgrounds containing the wild-type, D1082G, or R376W forms of *PDR1*. First, we tested these strains for their fluconazole susceptibility. The loss of Cna1 from either GOF-expressing form of Pdr1 caused an increase in azole susceptibility, although a considerable reduction of susceptibility was maintained (Fig. 2A). We also assessed the effect of a *cna1*Δ allele on the level of wild-type and fluconazole-induced *CDR1* and *PDR1* expression at both the RNA and protein level (Fig. 2B, C, D, and F). In all cases, loss of Cna1 reduced expression of both *CDR1* and *PDR1*. This reduction correlated well with the observed increase in fluconazole susceptibility of these GOF strains lacking calcineurin and argues that calcineurin activity is required for full function of even these GOF forms of Pdr1.

These findings are consistent with calcineurin acting as an upstream modulator of susceptibility to two of the most important classes of antifungal drugs: azoles and echinocandins. The mechanism of calcineurin activation of Crz1 for echinocandin susceptibility (3) and Pdr1 for fluconazole susceptibility seems to be different, at least genetically, since a hyperactive allele of *CNA1* leads to echinocandin susceptibility that is lower than that seen in the wild-type strain, while this same allele simply restores azole susceptibility to normal (Fig. 1; Fig. S2). We also provide an explanation for the previously observed increase in fluconazole susceptibility of a *cna1*Δ strain (4) as this strain exhibits a defect in the expression of both *CDR1* and *PDR1* under wild-type and fluconazole-challenged conditions. Calcineurin is also required for the full function of two different GOF alleles of *PDR1*, a finding that indicates the increased activity of these transcription factors must involve more than a single positive input. The GOF forms of Pdr1 maintain significant expression of both Pdr1 itself and also the *CDR1* target gene in the absence of Cna1. While we see the association of Cna1 with Pdr1 in co-immunoprecipitation assays, we do not yet know if this interaction is direct. The mechanism underlying control of Pdr1 function by calcineurin catalytic activity is a main focus of future experiments. Our preliminary evidence suggests that bulk phosphorylation of Pdr1 is increased upon fluconazole challenge (data not shown) which suggests that the direct calcineurin target may be another factor, but this remains to be determined. Calcineurin is known to be stimulated by elevated Ca^2+^ levels in the cell and has been previously shown to impact fluconazole susceptibility (4). Others have found that calcium levels can increase upon fluconazole treatment, providing a potential link between calcineurin activation and Pdr1 (12). Fluconazole is fungistatic on *C. glabrata* but can be converted to fungicidal if combined with calcineurin inhibitors (13). We speculate that part of this conversion may involve inhibition of the calcineurin-dependent activation of the Pdr1/*CDR1* fluconazole resistance axis we describe here.

## SUPPLEMENTAL MATERIAL

Supplemental material mbio.02416-23-s0001.pdf Supplemental figures and Materials and Methods.

ASM does not own the copyrights to Supplemental Material that may be linked to, or accessed through, an article. The authors have granted ASM a non-exclusive, world-wide license to publish the Supplemental Material files. Please contact the corresponding author directly for reuse.
