# Supplementary material for: Calcineurin is required for Candida glabrata Pdr1 transcriptional activation
Source: mBio. 2023 Nov 9;14(6):e02416-23. doi: 10.1128/mbio.02416-23 (PMC10746151; doi:10.1128/mbio.02416-23)

Supplementary Figures and Material and Methods

Supplementary Figure 1. **Co-immunoprecipitation of Pdr1 with epitope-tagged Cna1**. Transformants expressing various forms of *PDR1* and the low-copy-number *CNA1*-2X FLAG-tagged allele were grown to mid-log phase, treated with 20 μg/ml of fluconazole for 2 hours at 30°C. Whole cell protein extracts were then prepared under nondenaturing conditions and subjected to immunoprecipitation using anti-Pdr1 antibody to recover Pdr1 and associated proteins. A fraction (1%) of the total extract was reserved to serve as an input control (Input) with the remainder used for anti-Pdr1 immunoprecipitation. Immunoprecipitates were washed and then separated on SDS-PAGE followed by western blotting analysis using polyclonal anti-Pdr1 and mouse monoclonal anti-FLAG antisera. Location of the proteins of interest is indicated by the arrows.

Supplementary Figure 2. **Western blot analyses of the role of Cna1 in expression of Pdr1 target genes**. A. Isogenic wild-type and *cna1Δ* strains were grown to mid-log phase and then challenged with 20 μg/ml fluconazole for the times indicated as described in Figure 1A and 1B. Whole cell protein extracts were prepared under denaturing conditions were prepared and analyzed by western blotting using polyclonal antisera detecting Cdr1 or Pdr1, an anti-peptide antibody detecting Erg11 or a mouse monoclonal against tubulin. The transferred proteins were stained with Ponceau S on the membrane to ensure equivalent transfer and loading. B. Quantitation of the western blotting in part A is shown. C. Western blotting of different proteins of interest as described in Figure 1C and 1D. Note the equivalent levels of the Cna1-2X HA proteins indicating that the defects caused by the catalytic mutant form of Cna1 (H190A) are not

due to lack of expression of proteins containing this mutation. D. Quantitation of the expression of the indicated proteins in panel C is shown.

Supplementary Figure 3. **Defective Pdr1 regulation is reproduced by loss of the calcineurin regulatory subunit but not a different protein phosphatase.** A. Isogenic strains lacking either the calcineurin regulatory subunit-encoding gene (*CNB1*) or a different serine/threonine protein phosphatase (*PPT1*) were tested for resistance to the indicated concentration of fluconazole as described previously. B. Expression of the indicated proteins was evaluated by western blotting as above. C. Quantitation of the western blot assay in B is shown.

Supplementary Figure 4. **Chemical inhibition of calcineurin activity phenocopies genetic loss of the phosphatase.** A. Isogenic wild-type and *cna1Δ* strains were analyzed by serial dilution on YPD medium containing the indicated drugs. FK506 is an inhibitor of calcineurin function and was added alone or in combination with caspofungin or fluconazole. B. Wild-type cells were grown to mid-log phase and then treated with FK506, fluconazole or both drugs. After two hours, *PDR1* and *ERG11* mRNA levels were assessed by RT-qPCR assay. C. Western blot analysis of cells treated as above using the indicated antisera. D. Quantitation of the western blot data in C.

Materials and methods

Reagents, *C. glabrata* strains and growth conditions. Fluconazole was purchased from LKT (laboratories, St Paul, MN). Caspofungin was purchased from Apexbio (Houston, TX). FK506 was purchased from Enzo Life Sciences (Farmingdale, NY). General growth conditions, nourseothricin section, and recyclable marker eviction

techniques were previously described in (1). All strains used in this study are listed in the supplemental table 1. Resistance phenotypes were assayed by plating serial dilutions of log phase cultures on solid media. Transformations were carried out using a standard lithium acetate protocol.

Strain and plasmid construction.

CNA1, *CNB1*, and *PPT1* gene deletion constructs were made by assembling the recyclable cassette from pBV65 (1) and fragments from the immediate upstream/ downstream regions of each gene by Gibson assembly cloning (New England Biolabs, Ipswich, MA). Eviction of the recyclable cassette left a single copy of *loxP* in place of the removed target gene coding region. Standard homologous transformation was used and all disruption mutations were verified by PCR using appropriate primers (Supplemental table 2).

The 3X HA epitope was inserted into the C-terminus of the *CNA1* gene in place of the native stop codon. The *CNA1*-3X HA cassette was cloned into the pBV133 vector (2), which carries a nourseothricin selection maker. From this plasmid, carrying the wildtype version of *CNA1*-3X HA, *CNA1*-H190A-3X HA, *CNA1*-AID-3X HA, and *CNA1*-AID-H190A-3X HA plasmids were subsequently generated from Gibson assembly cloning (New England Biolabs).

To clone the *CNA1* allele as a C-terminal fusion with a 2X FLAG tag, the low-copy-number vector pCnat-*CNA1*-3X HA described above was linearized with the restriction enzymes SacI and KpnI to replace the 3X HA tag with 2X FLAG. The 2X FLAG sequence was introduced via PCR as an oligonucleotide primer along with a reverse primer that produced the 3' *CNA1* UTR and cloned by use of the Gibson

Assembly Cloning Kit (NEB #E5510S). The identity of the vector pCnat-*CNA1*-2X FLAG was also confirmed by sequencing.

Quantification of transcript levels by RT-qPCR, antibodies and western immunoblot analysis. Detailed protocols were previously described (2-4). Target gene transcript levels were normalized to transcript levels of 18S rRNA. The HA antibody (clone 2-2.2.14) was purchased from Invitrogen (Carlsbad, CA). The Pdr1, Cdr1, and Erg11 antibodies were previously described (3-5).

Statistics. All assays were done at least as biological replicates with a minimum of two technical replicates. The Student T-test was used to assess the statistical significance of results of comparisons of samples. Unpaired conditions were used throughout with the exception of Supplementary Figure 4 in which paired conditions were applied. (*, P < 0.5; **, P < 0.01; ***, P < 0.001).

Literature cited

1. Vu BG, Moye-Rowley WS. 2018. Construction and Use of a Recyclable Marker To Examine the Role of Major Facilitator Superfamily Protein Members in Candida glabrata Drug Resistance Phenotypes. mSphere 3.
2. Vu BG, Stamnes MA, Li Y, Rogers PD, Moye-Rowley WS. 2021. The Candida glabrata Upc2A transcription factor is a global regulator of antifungal drug resistance pathways. PLoS Genet 17:e1009582.

3. Vu BG, Moye-Rowley WS. 2022. Azole-Resistant Alleles of ERG11 in Candida glabrata Trigger Activation of the Pdr1 and Upc2A Transcription Factors. Antimicrob Agents Chemother 66:e0209821.

4. Vu BG, Thomas GH, Moye-Rowley WS. 2019. Evidence that Ergosterol Biosynthesis Modulates Activity of the Pdr1 Transcription Factor in Candida glabrata. MBio 10.

5. Paul S, Bair TB, Moye-Rowley WS. 2014. Identification of Genomic Binding Sites for Candida glabrata Pdr1 Transcription Factor in Wild-Type and rho0 Cells. Antimicrob Agents Chemother 58:6904-12.

Supplemental table 1: Strain list.

Strain name	Parent	Genotype	Reference
CBS138 (ATCC2001)	CBS13 (ATCC2001)	Wild-type isolate	ATCC
BVGC65	CBS138	*pdr1Δ::loxP*	(1)
BVGC379	CBS138	*cna1Δ::loxP*	This study
BVGC600	CBS138	*cnb1Δ:: loxP*	This study
BVGC612	CBS138	*ppt1Δ:: loxP*	This study
BVGC84	BVGC65	*PDR1::loxP*	(2)
BVGC86	BVGC65	D1082G *PDR1::loxP*	(2)
BVGC87	BVGC65	R376W *PDR1::loxP*	(2)

BVGC390	BVGC84	*PDR1::loxP cna1Δ::loxP*	This study
BVGC394	BVGC86	D1082G *PDR1::loxP* *cna1Δ::loxP*	This study
BVGC397	BVGC87	R376W *PDR1::loxP* *cna1Δ::loxP*	This study

<u>Supplemental table 2: Primer list.</u>

CNA1 Forward: GTGCAACTAGCGAAGGACAC
CNA1 Reverse: CAGAAATATGGCGATGGTCAGTCAC

CNB1 Forward: CAGGGACATCGTACATATCATGC
CNB1 Reverse: GTACGCTCATCGATCACATTATCCTC

PPT1 Forward: CTTTCGTCTCGGAGAAAGCG
PPT1 Reverse: CGAACTGAAAGGTCATCACTGC

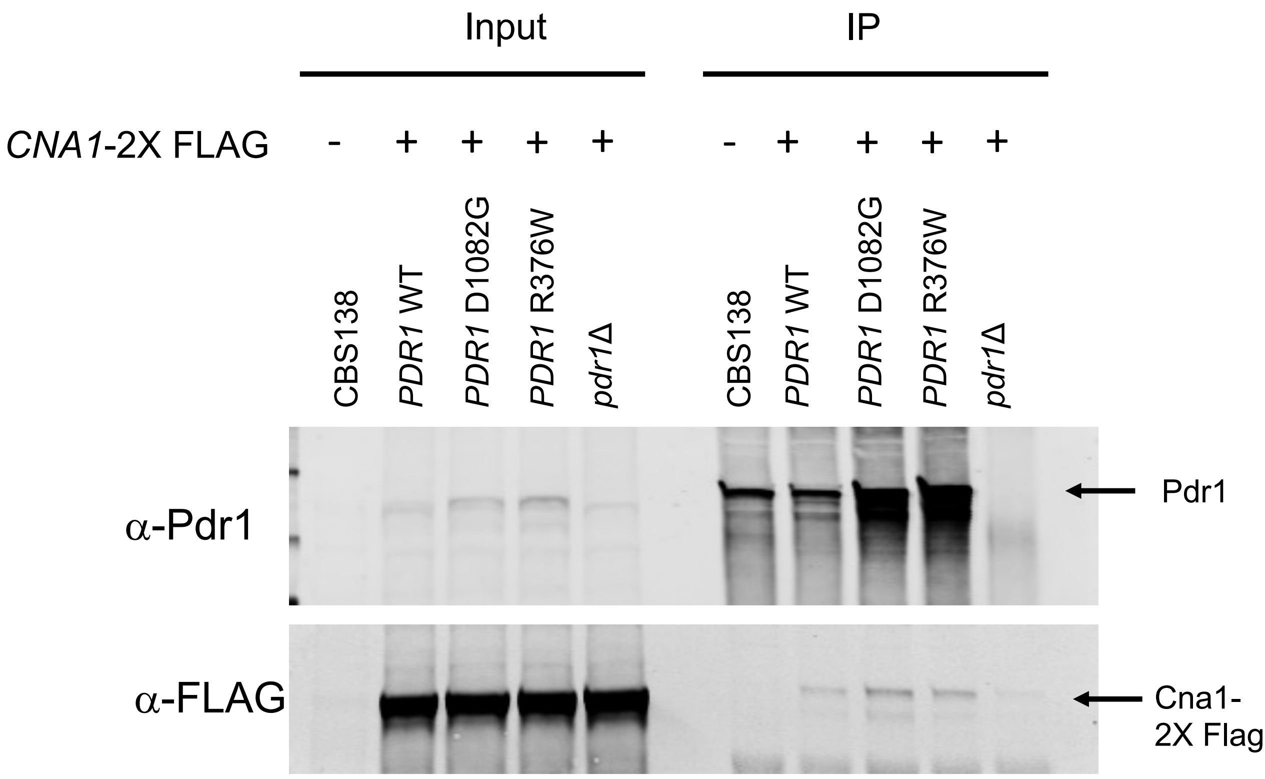
Input
IP
CNA1-2X FLAG
- + + + +
- + + + +
CBS138
PDR1 WT
PDR1 D1082G
PDR1 R376W
pdr1Δ
CBS138
PDR1 WT
PDR1 D1082G
PDR1 R376W
pdr1Δ
α-Pdr1
α-FLAG
Pdr1
Cna1-
2X Flag

Supplementary Figure 2. Vu, et al.

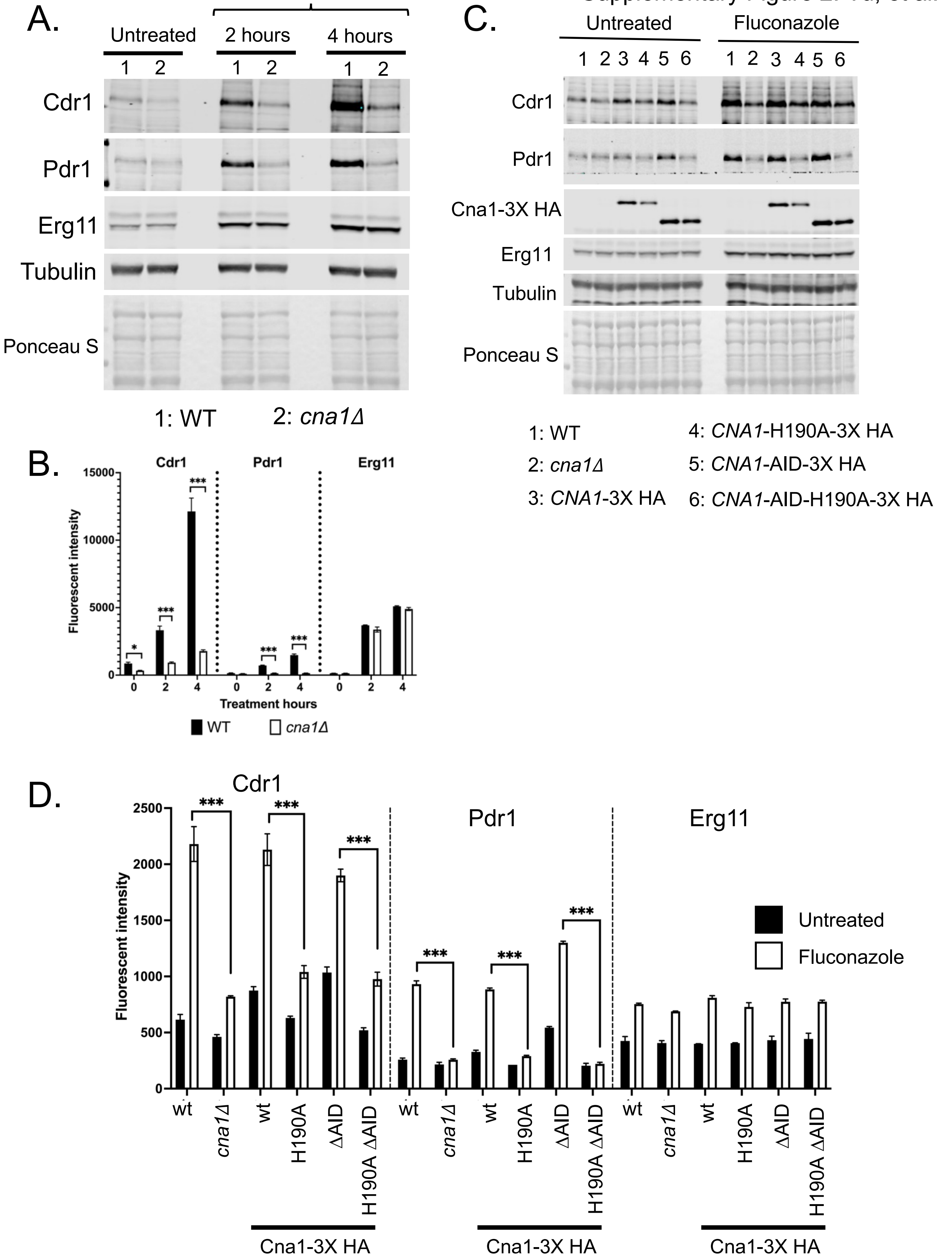

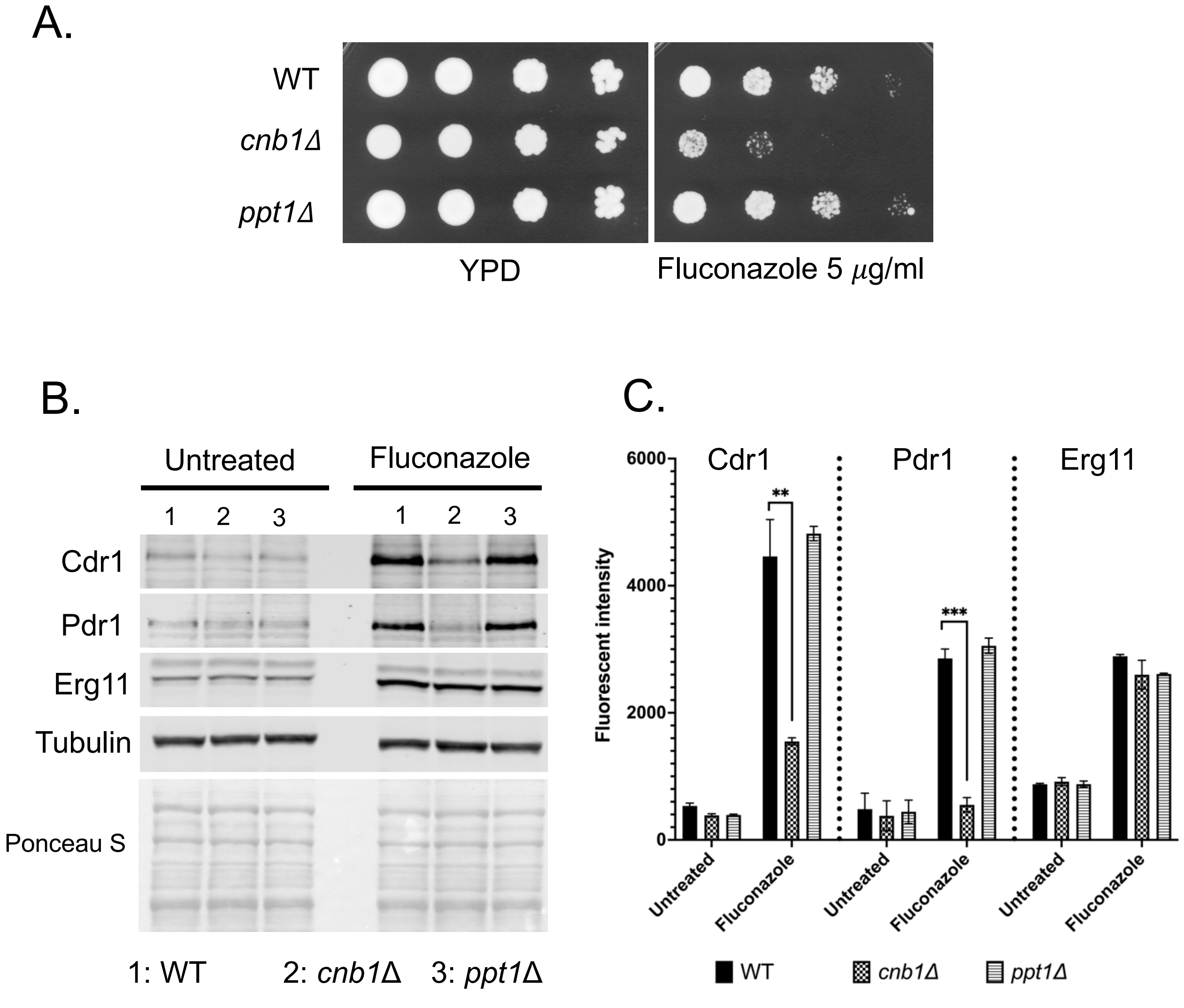
A.
WT
cnb1Δ
ppt1Δ
YPD
Fluconazole 5 μg/ml
B.
Untreated
Fluconazole
1 2 3
1 2 3
Cdr1
Pdr1
Erg11
Tubulin
Ponceau S
1: WT
2: cnb1Δ
3: ppt1Δ
C.
Cdr1
Pdr1
Erg11
Fluorescent intensity
6000
4000
2000
0
**
***
Untreated
Fluconazole
WT
cnb1Δ
ppt1Δ

Supplementary Figure 4. Vu, et al.

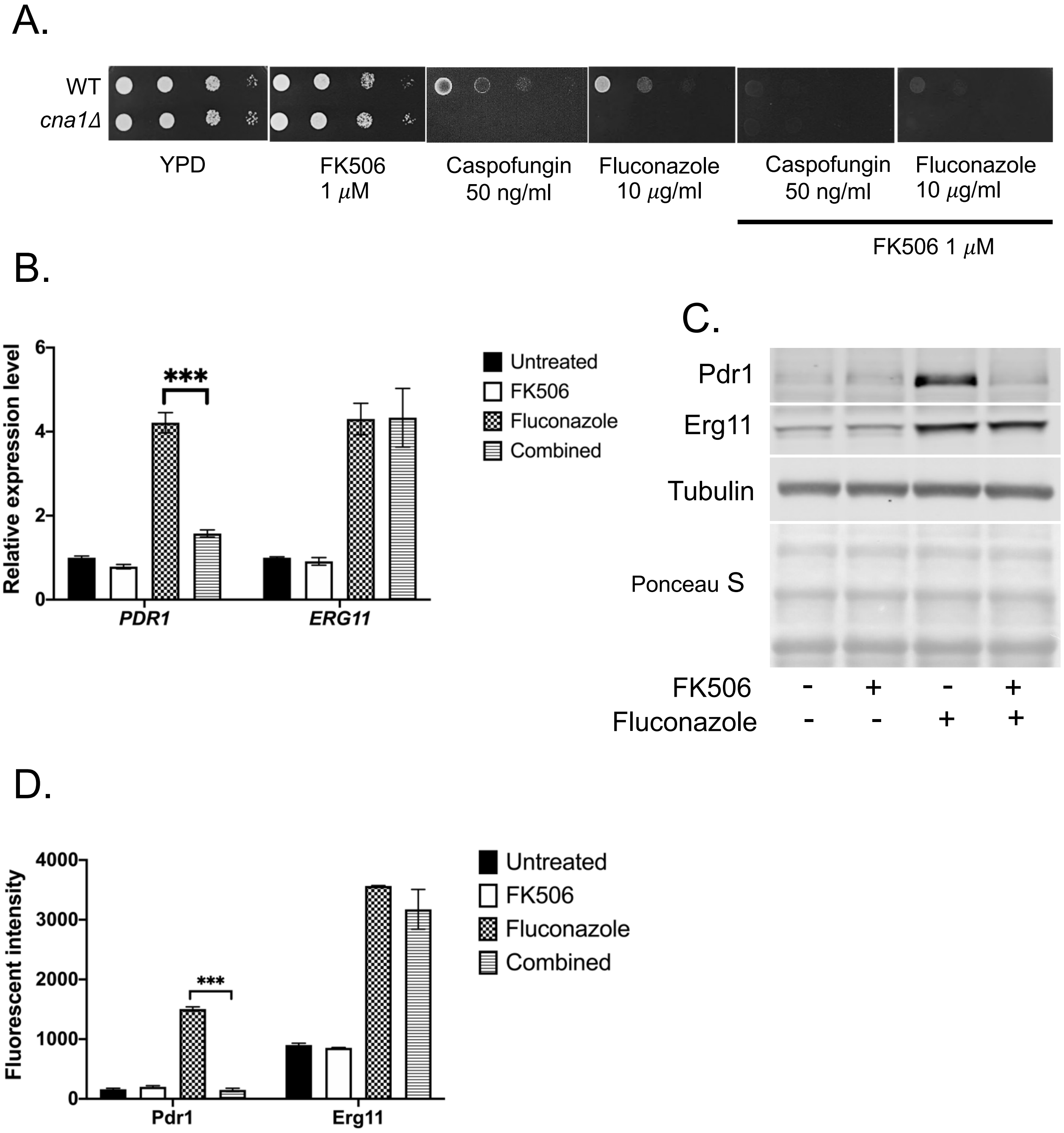

Supplement: Supplemental material — Supplemental figures and Materials and Methods. [file mbio.02416-23-s0001.pdf]
